# Birth Satisfaction and Breastfeeding Attitudes Among Mothers Aged 35 and Older

**DOI:** 10.3390/nu17233796

**Published:** 2025-12-03

**Authors:** Amelia Julia Sobala, Anna Weronika Szablewska

**Affiliations:** Department of Obstetric and Gynecological Nursing, Institute of Nursing and Midwifery, Gdansk Medical University, Sklodowskiej-Curie 3 A, 80-210 Gdansk, Poland

**Keywords:** birth satisfaction, attitudes towards breastfeeding, late motherhood, social support, perinatal care, psychosocial factors

## Abstract

**Background/Objectives:** Delayed motherhood is becoming increasingly common, yet limited evidence exists on birth satisfaction and breastfeeding attitudes among women aged ≥35. In this study, the hypothesis was tested whether higher birth satisfaction and stronger social support are associated with more positive breastfeeding attitudes and if previous childbirth experience moderates these relationships. **Methods:** A cross-sectional online survey was conducted among 148 Polish women up to 12 months postpartum. Participants were recruited via social media and parenting-related online communities; the survey was disseminated across multiple online channels to enhance representativeness and reduce potential sampling bias. Eligibility included age ≥35 at childbirth and informed consent. The sample size was considered adequate based on recommendations for regression models with the number of included predictors. Validated tools were used (MSPSS, IIFAS-Pol, BSS-R PL). Assumptions for Pearson’s correlation coefficients and linear regression (normality, homoscedasticity, absence of multicollinearity) were checked prior to analyses. The analysis was performed using IBM SPSS Statistics 29 (IBM Corp., Armonk, New York, NY, USA). **Results:** Women aged ≥35 reported high birth satisfaction and generally positive breastfeeding attitudes. Among multiparous women, birth satisfaction was moderately positively associated with breastfeeding attitudes (BSS-R PL; r = 0.396, *p* < 0.01), and perceived social support showed a small but significant association (MSPSS; r = 0.249, *p* < 0.05). Hierarchical regression analyses indicated that, in women over 35 with a subsequent child, psychosocial variables significantly predicted breastfeeding attitudes, whereas the control model—including education, socioeconomic status, and delivery mode—was not significant. Adding birth satisfaction and perceived social support improved model fit (*R*^2^ = 0.194), with birth satisfaction emerging as the only significant predictor (β = 0.31). The corresponding effect size (*f*^2^ = 0.143) indicated a near-medium effect. In contrast, neither the control nor the extended model was significant among primiparous women, suggesting no meaningful associations in this group. **Conclusions:** Higher birth satisfaction and perceived social support may promote more favorable breastfeeding attitudes in women becoming mothers at ≥35, with stronger effects among multiparous mothers. These findings highlight the need for individualized prenatal education, enhanced psychosocial support, partner involvement and efforts to improve childbirth experiences. Interventions tailored to women entering motherhood at an advanced maternal age are warranted.

## 1. Introduction

Late motherhood is an increasingly popular trend observed in both highly and moderately developed countries [1,2,3]. According to most authors, the term “late motherhood” is defined as becoming a mother at the age of 35 years or older [2,3,4,5]. With the introduction of contraceptives, the expanding opportunities for women’s educational and professional development, and economic uncertainty in the global market, decisions regarding motherhood are increasingly postponed. The desire to achieve educational, professional and financial stability consequently leads to a steady increase in the average age of women giving birth to their first child and a simultaneous decline in birth rates. The continuous advancement of modern, assisted reproductive technologies reinforces the societal belief that reproductive plans can be safely postponed [3,4,5].

Along with the growing trend of delaying motherhood come concerns about the potential health consequences and well-being of both mothers and children. Pregnancy in older women carries a higher risk of complications, especially pregnancy-induced hypertension and gestational diabetes [3,6,7]. Among this group of women, a higher rate of cesarean sections is also observed [3,4,6]. Advanced maternal age is further associated with an increased risk of miscarriages and genetic abnormalities [3]. In several studies, a higher likelihood is indicated of pre-term birth and intrauterine fetal death [2,3,8,9]. The risks associated with advanced maternal age mean that pregnancy in women over 35 is classified as a high-risk pregnancy (Regulation of the Polish Minister of Health, 11 September 2018).

At the same time, many authors point out that maternal age does not have significant impact on the newborn’s state; for example, advanced maternal age has not been found to notably affect the incidence of respiratory disorders, low Apgar scores or birth asphyxia in newborns [3,6]. Although considerable evidence is provided in medical and demographic literature underlining that pregnancy at an advanced age may be associated with an increased risk of complications (e.g., pre-term birth, pregnancy-induced hypertension, gestational diabetes, high cesarean section rates and increased somatic morbidity among mothers) [3,6], in a growing body of research, psychosocial benefits of delayed motherhood are also highlighted. In scoping reviews, it is indicated that older mothers may exhibit greater emotional maturity, a higher level of readiness for motherhood and a more deliberate parenting style [10].

Beyond medical risks, late motherhood is increasingly recognized as a multifaceted experience shaped not only by medical circumstances but also by distinct psychosocial characteristics. Nevertheless, a clear research gap persists regarding maternal well-being, birth satisfaction and breastfeeding experiences among women of advanced maternal age. Understanding these psychosocial dimensions is essential for informing perinatal interventions and public health strategies aimed at supporting maternal well-being.

Prior studies indicate that women aged ≥35 perceive pregnancy and childbirth as higher-stakes events and report heightened risk awareness, emotional tension and stronger anticipatory concerns compared with younger mothers, all of which may influence their appraisal of birth experiences [11,12]. Age and parity have also been associated with differences in pregnancy-related anxiety and maternal self-efficacy: older first-time mothers often experience greater uncertainty, whereas multiparous women may draw on prior experience to regulate emotions and form more stable expectations [12]. Perceived social support constitutes a key factor in this context, as it is consistently linked to higher maternal self-efficacy, lower depressive symptoms [13] and more favorable breastfeeding behaviors [14]. Social support may therefore influence how women interpret childbirth and translate their experiences into infant-feeding attitudes. Although previous literature has suggested potential mediating or moderating roles of social support, these mechanisms have not been formally tested in the present study.

An expanding body of research indicates that psychosocial mechanisms play a central role in shaping perinatal experiences among women of advanced maternal age. Two complementary theoretical frameworks—the Expectancy–Value Model (EVM) and the Stress and Coping Theory—provide a coherent basis for understanding the relationships examined in this study.

According to the EVM, attitudes and behavioral intentions arise from the interaction between the subjective value attributed to a behavior and the expectancy of successfully performing it. In the perinatal context, these cognitive–motivational mechanisms shape how women evaluate childbirth experiences and develop subsequent attitudes toward infant feeding. A positive birth experience may reinforce perceived control and strengthen both value and expectancy components, whereas discrepancies between expectations and the actual course of childbirth may reduce maternal confidence and undermine motivation. Classic work by Janke and by Avery et al. demonstrated that women who discontinued breastfeeding earlier than planned often held strong initial intentions, suggesting later disruptions in perceived expectancy or value—an observation consistent with EVM principles [15].

The Stress and Coping Theory complements this perspective by explaining how women appraise and respond to perinatal stressors. Coping responses depend on perceived situational demands and available internal and external resources. Social support is a central protective factor within this framework. Evidence from Ribeiro et al. shows that positive social support enhances adaptive coping and facilitates the transition to motherhood [16], while findings by Ma et al. indicate that emotional support—particularly from a spouse—buffers the impact of perinatal stressors [17].

Integrating the EVM and the Stress and Coping Theory offers a robust theoretical rationale for the present study. While the EVM elucidates how cognitive evaluations—such as birth satisfaction—may influence attitudes toward breastfeeding, the Stress and Coping Theory explains why social support and coping resources may strengthen or attenuate these evaluative pathways. This combined perspective is particularly relevant for older mothers, who may enter childbirth with heightened expectations, increased risk awareness and substantial reliance on supportive relationships [18,19,20].

When integrated, these frameworks suggest that higher birth satisfaction and stronger social support should be associated with more positive breastfeeding attitudes, and that these associations may vary by parity. Multiparous women, who possess more established reference points and coping schemas, may show stronger links between evaluative processes and feeding attitudes than first-time mothers of advanced maternal age. These theoretical considerations informed the hypotheses of the present study, which examines relationships between birth satisfaction, perceived social support and breastfeeding attitudes among primiparous and multiparous women aged ≥35.

Therefore, the aim of the present study is to fill this gap by evaluating birth satisfaction and attitudes towards breastfeeding among women who chose to become mothers after the age of 35. In this analysis, differences are captured in the psychosocial experiences associated with late motherhood and factors, such as social support and previous perinatal experiences, which may influence both satisfaction and breastfeeding success are identified. In the study, the authors seek to contribute to the existing literature on the psychosocial aspects of late motherhood, and its findings may serve to inform the planning and improvement of perinatal care.

## 2. Materials and Methods

### 2.1. Study Design

The present research is a cross-sectional study conducted among 148 Polish women up to 12 months postpartum. In Poland, ethnic minorities constitute a relatively small share of the total population; thus, no distinction was made between ethnic groups. The study design was planned and described in accordance with STROBE guidelines for cross-sectional studies [21]. All procedures were conducted in accordance with the principles of the 1964 Declaration of Helsinki of the World Medical Association (WMA) concerning research involving human participants and were approved by the Bioethics Committee of the Medical University of Gdańsk (Approval No. KB/16/2026).

### 2.2. Study Setting

The study was carried out using an online self-administered diagnostic survey completed by the participants independently. The data collection and participant recruitment period lasted from March 2025 to September 2025.

### 2.3. Participants

Before beginning the questionnaire, participants were given detailed information on the study and provided electronic, informed consent for participation. Open online recruitment was used. A total of n = 148 women were included in the analysis, divided into two groups:

- Group 1 (primipara ≥35 years): women who gave birth to their first child at age ≥35 (n = 81),
- Group 2 (multipara ≥35 years): women who gave birth to a subsequent child at age ≥35 (n = 67).

### 2.4. Inclusion and Exclusion Criteria

Participants were eligible for inclusion if they met the following criteria: 35 years or older at the time of childbirth (either their first or subsequent child), within 12 months postpartum, proving informed consent to participate and ability to complete the questionnaire independently in Polish. Exclusion criteria included: multiple pregnancies, cognitive impairments that could hinder independent participation and language barriers preventing comprehension of the survey content. This approach ensured that the study group was homogeneous in terms of postpartum status and cognitive ability, while minimizing potential confounding factors related to communication difficulties or complex obstetric outcomes associated with multiple pregnancies.

### 2.5. Data Collection Tools

The questionnaire consisted of seven sections, presented in the following order:

Sociodemographic data—including age, education, marital status and place of residence. Obstetric data—including number of pregnancies/childbirths, gestational age at delivery, mode of delivery, complications in the most recent pregnancy and use of assisted reproductive techniques.

Postpartum functioning—author-designed questions regarding relationships and support from close ones, as well as subjective functioning before and after delivery.

Breastfeeding—author-designed questions on initiation, difficulties and duration of exclusive breastfeeding.

Multidimensional Scale of Perceived Social Support (MSPSS; Polish adaptation by Buszman & Przybyła-Basista)—12 items rated on a 1–7 scale; a total score and three subscales (Family, Friends, Significant Other) were calculated. Higher scores indicate greater perceived social support [22]. In this study, the mean scores were 19.40 (SD = 7.91) for support from friends, 20.30 (SD = 7.06) for support from family, and 23.06 (SD = 6.68) for support from a significant other. The total MSPSS score totaled a mean of 62.76 (SD = 19.17), with individual subscale scores ranging from 4.00 to 28.00 and total scores ranging from 12.00 to 84.00. Median values were 21.00 for friends, 22.00 for family, 26.00 for a significant other, and 69.00 for the total score. Cronbach’s alpha for the MSPSS was 0.958.

Iowa Infant Feeding Attitude Scale (IIFAS; Polish adaptation by A. Bień)—16 items rated on a 1–5 scale; a total score was calculated after reversing specified items according to the manual. Higher scores indicate a more positive attitude towards breastfeeding [23]. In this study, attitudes towards breastfeeding yielded a mean of 30.51 (SD = 6.31), a median of 31.50, and scores ranging from 16 to 40. For attitudes towards formula feeding, a mean of 31.17 (SD = 5.71) was obtained, a median of 32.00, and a range from 18 to 40. The total IIFAS-Pol score showed a mean of 61.68 (SD = 10.91), a median of 64.00, with a minimum of 34 and a maximum of 80. The IIFAS-Pol demonstrated good internal consistency, with Cronbach’s alpha at 0.838.

Revised Birth Satisfaction Scale-BSS-R PL (Polish adaptation by Pawlicka, Wróbel, Baranowska, Macewicz)—10 items rated on a 0–4 scale; a total score (0–40) and three subscales were calculated: Quality of Care (QOC), Women’s Attributes (WBA), and Experienced Stress (SL). Higher scores indicate greater birth satisfaction [24]. In this study, the mean score was 24.14 (SD = 6.86), and the median of 25.00 was achieved. Scores ranged from 4.00 to 37.00. The BSS-R PL demonstrated good internal consistency, with Cronbach’s alpha at 0.824.

In the study, a fixed order of seven questionnaire sections was adopted, beginning with sociodemographic and obstetric items, followed by more personal questions on postpartum functioning and infant feeding, and concluding with three standardized psychometric scales. This structure likely reduced the risk of bias, as emotionally demanding content did not appear at the outset, and psychometric instruments were administered only after establishing the contextual background. Nevertheless, potential order effects cannot be entirely ruled out, as earlier reflections on postpartum challenges may have influenced responses on the BSS-R and IIFAS scales.

Additionally, the average completion time of approximately 20 min may have introduced respondent fatigue, potentially reducing the precision of answers in the final sections. Given the relatively brief and validated nature of the instruments used, this risk should be considered moderate and acceptable.

### 2.6. Sample Size

In the study, two groups of mothers were compared, as given in the Section 2.3. Although no formal a priori sample size calculation was carried out, the final sample allows for adequately powered comparative analyses. With the obtained group sizes (81 vs. 67) and a two-tailed significance level of α = 0.05, the study has approximately 80% power to detect small-to-moderate effect sizes, corresponding to Cohen’s d ≈ 0.45 for between-group comparisons. This level of sensitivity is appropriate for cross-sectional comparative research aiming to identify meaningful differences between groups of women differing by parity at advanced maternal age. Furthermore, the total sample size (n = 148) meets commonly accepted recommendations for observational studies and provides sufficient statistical stability for multivariable analyses conducted within the study. Overall, the sample size can be considered adequate to detect small-to-moderate effects in between-group comparisons and to perform the planned multivariable analyses.

### 2.7. Bias

To minimize recall bias, women were included no later than 12 months postpartum. Multiple pregnancies were excluded to reduce the influence of specific obstetric complications and to ensure a more homogeneous sample. We acknowledge that the use of online recruitment may have led to an overrepresentation of women with higher education and greater digital literacy. In our sample, the proportion of women with university-level education was high (73.1% of multiparous and 82.7% of primiparous mothers), which may limit the generalizability of the findings to the broader population of mothers aged ≥35. Generalizations should therefore be made with caution.

### 2.8. Statistical Analysis

The collected data were analyzed using IBM SPSS Statistics, version 29. Analyses were conducted to assess differences and relationships between groups of mothers aged above 35. Relationships between continuous variables were examined using Pearson’s correlation coefficient, which is used to evaluate the strength and direction of linear associations. Associations between categorical variables, such as type of feeding or breastfeeding experience and maternal status (primipara vs. multipara), were estimated using the chi-square (χ^2^) test of independence. Additionally, linear regression analysis was conducted to identify the variables that significantly influence attitudes towards breastfeeding (IIFAS-Pol). Normality evaluation of all measured variables in both groups indicated that the distributions were acceptable for parametric analysis. The values of skewness and kurtosis for all scales fell within commonly accepted thresholds for approximately normal distributions (i.e., between −1 and +1). The majority variables showed slight negative skewness, reflecting a mild shift toward higher scores, while kurtosis values were close to zero, suggesting neither excessive peakedness nor flattening of the distributions. Given the sample sizes of the two groups (n = 67 and n = 81), and considering the robustness of parametric tests with samples above 30, the distributions can be considered sufficiently normal for comparative statistical analyses. We have assessed the assumption of multicollinearity using VIF (Variance Inflation Factor) statistics. In both regression models, VIF values ranged from 1.032 to 1.377. These results are well within accepted thresholds (Tolerance > 0.10, VIF < 10), indicating that no multicollinearity is present between the predictors. The scatterplots displaying standardized residuals against standardized predicted values show no strong indication of heteroscedasticity. For the significant regression model, effect sizes were calculated using the $f^{2}$ statistic, which is based on changes regarding the coefficient of determination ($R^{2}$) in regression models. Specifically, $f^{2}$ is computed as $f^{2}=R_{\mathrm{inc}}^{2}/(1-R_{\mathrm{inc}}^{2})$, where $R_{\mathrm{inc}}^{2}\;$ represents the increase in the overall $R^{2}\;$ when a specific predictor is added to a model that already includes other predictors. This measure reflects the unique contribution of the predictor to the explained variance. A rule of thumb is that for the $f^{2}\;$ statistic, 0.02 is a small effect, 0.15 is a medium effect, and 0.35 is a large effect.

Descriptive statistics were also calculated: for continuous variables, means, standard deviations, medians, minimum and maximum values; for categorical variables, frequencies and percentages were estimated. Statistical significance was set at *p* < 0.05.

## 3. Results

### 3.1. Characteristics of the Study Group

Sociodemographic and obstetric characteristics are presented in Table 1.

### 3.2. Analysis of Data Related to Breastfeeding and Birth Satisfaction

**Figure 1:**
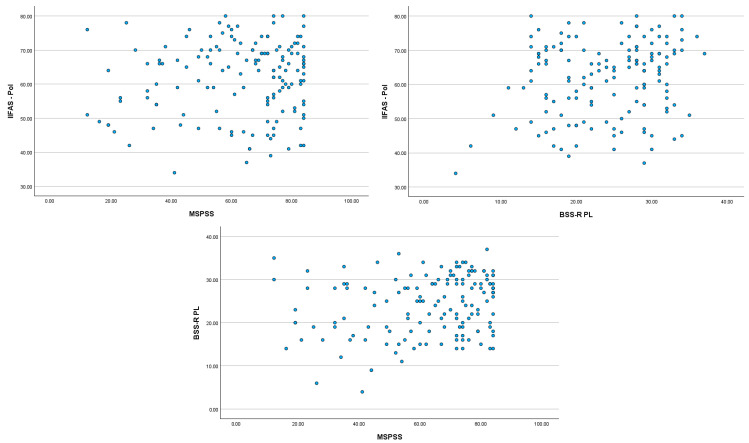
Results of correlation analysis between positive attitudes towards breastfeeding and formula feeding, IIFAS-Pol score, MSPSS and BSS-R PL in the group of mothers aged >35 years, subsequent child.

The sociodemographic and obstetric characteristics of the study population are presented in Table 1. Women aged ≥35 years reported high levels of birth satisfaction and generally positive attitudes toward breastfeeding. Exclusive breastfeeding was the most common feeding method in both groups, with slightly higher prevalence among multiparous women (68.7%, n = 46) compared with primiparous women (61.7%, n = 50). Positive breastfeeding experiences were also more frequently reported by multiparas (71.6%, n = 48) than by primiparas (53.1%, n = 43), as shown in Table 1. Correlation analyses revealed clear parity-related differences. Among multiparous women, birth satisfaction showed a moderate positive association with breastfeeding attitudes (BSS-R PL; r = 0.396, *p* < 0.01), and perceived social support demonstrated a small but statistically significant association (MSPSS; r = 0.249, *p* < 0.05). Birth satisfaction was also correlated with social support in this group. These associations are presented in Table 2 and visualized in Figure 1. In primiparous women, none of the correlations between breastfeeding attitudes, birth satisfaction, or perceived social support reached statistical significance.

Hierarchical multiple regression analyses were conducted to examine the influence of perceived social support (MSPSS) and birth satisfaction on IIFAS-Pol scores among women over 35, controlling for education, socioeconomic status and delivery mode. For women having a subsequent child, the model including only control variables was not significant (F(3, 63) = 1.558, *p* = 0.208), whereas adding MSPSS and birth satisfaction produced a significant model (F(5, 61) = 2.927, *p* = 0.020). Among primiparous women, neither the control model nor the extended model reached statistical significance, and none of the predictors contributed meaningfully to breastfeeding attitudes (Table 3). Overall, psychosocial variables were associated with breastfeeding attitudes only among multiparous women, whereas such associations were not observed in first-time mothers aged ≥35 years.

Regression analyses further confirmed this pattern. In multiparous women, the control model—comprising education, economic status and mode of delivery—was not statistically significant. In contrast, the model including birth satisfaction and social support significantly explained breastfeeding attitudes (*R*^2^ = 0.194; adjusted *R*^2^ = 0.127). Birth satisfaction was the only significant predictor (β = 0.31, *p* = 0.025), accounting for an additional 12.4% of explained variance. The corresponding effect size (*f*^2^ = 0.143) indicated a near-medium effect. Perceived social support did not independently predict breastfeeding attitudes after adjustment for covariates. Full regression coefficients are provided in Table 3. A near-medium effect suggests that improving birth satisfaction may have a meaningful impact on breastfeeding attitudes, which is particularly relevant when designing childbirth support interventions or programs promoting breastfeeding. Enhancing birth satisfaction through personalized care and emotional support may promote more positive breastfeeding attitudes in multiparous women. For first-time mothers, since birth satisfaction and perceived social support did not predict breastfeeding attitudes, interventions such as targeted breastfeeding education, skill-building, and anticipatory guidance may be more effective. Overall, focusing on the psychosocial quality of the birth experience, rather than solely on demographic or delivery-related factors, appears crucial for encouraging positive breastfeeding attitudes.

## 4. Discussion

Our study provides important data regarding childbirth satisfaction and attitudes towards breastfeeding among women who became mothers after the age of 35. The findings demonstrate complex relationships between infant-feeding attitudes, perceived social support and birth satisfaction.

Although the IIFAS was originally developed to distinguish between more positive and negative attitudes towards breastfeeding, emerging research allow to suggest that infant-feeding attitudes among contemporary mothers are often multidimensional rather than strictly polarized. Instead of adhering to a single feeding ideology, many women adopt a flexible and pragmatic orientation to feeding, shaped by contextual needs, personal preferences and family dynamics. For example, mothers may recognize the emotional and health benefits of breastfeeding while also acknowledging the practicality, convenience or shared caregiving potential associated with formula feeding—an approach reflected in studies indicating that feeding decisions are frequently embedded in broader, situationally driven reasoning processes [25]. Similar insights are reported in qualitative and mixed-methods research, demonstrating that mothers evaluate feeding choices dynamically, taking social expectations, available support, lifestyle demands and personal well-being into account, rather than making decisions based solely on an exclusive commitment to one feeding method [26]. In a recent systematic review, it was further demonstrated that mixed-feeding practices can represent adaptive strategies responding to maternal autonomy, external pressures and changing circumstances, rather than signaling confusion or ambivalence autonomy [27]. Such findings align with the theoretical perspectives emphasizing responsive parenting and maternal subjectivity, in which feeding becomes part of a fluid, negotiated process aimed at balancing maternal well-being and infant needs [28].

Within this broader conceptual context, our results—based on overall attitudes towards infant feeding—may be viewed as consistent with the notion that women of advanced maternal age, approach feeding decisions in individualized and context-dependent ways, rather than doing so in accordance with a simple breastfeeding—formula feeding dichotomy. It is also important to interpret the associations between infant-feeding attitudes with some caution. Given the structure of the IIFAS-Pol, which includes reverse-coded and broadly evaluative items, part of the shared variance between breastfeeding and formula-feeding attitudes may reflect overlapping measurement content instead of fully distinct psychological constructs. Therefore, the observed associations should be viewed as indicative of general feeding orientations rather than strictly separate attitudinal dimensions.

A central result of the present study is that birth satisfaction predicted attitudes towards breastfeeding exclusively among multiparous women aged ≥35, which suggests that the relationship may differ according to parity. However, since moderation was not formally tested, this interpretation should be viewed as exploratory. In previous studies, it has been indicated that women who have given birth before typically exhibit higher maternal self-efficacy, greater emotional readiness and enhanced confidence in managing postpartum challenges [29]. Prior experience also contributes to more realistic expectations regarding the course of labor and the early breastfeeding period, thereby strengthening satisfaction and fostering more positive breastfeeding attitudes [30]. In contrast, primiparous women may experience higher levels of uncertainty and anxiety, which may attenuate the influence of birth satisfaction on feeding attitudes and can increase their susceptibility to negatively appraising stressful situations [31]. From the perspective of stress theory, previous experiences may serve as a “cognitive frame of reference”, facilitating the interpretation of current events and supporting more effective emotional regulation mechanisms.

Social support constitutes one of the essential psychosocial determinants of perinatal experiences and maternal adaptation. In the present study, particular attention was given to the relationship between social support and attitudes towards infant feeding among women aged 35 and older. The findings highlight that although social support—especially partner related—is associated with more positive breastfeeding attitudes, its role is not uniform and may differ depending on parity, the type of support available and the broader emotional context. According to the Stress and Coping Theory, social support functions as a buffering factor that mitigates stress associated with childbirth. Individuals who receive stronger emotional, practical, and informational support are better equipped to cope with the unpredictability of labor, which promotes a more positive appraisal of the childbirth experience. In our study, higher levels of social support were associated with greater birth satisfaction, confirming the mechanistic role of support as a resource that reduces perceived stress. This is consistent with previous literature indicating that support from partners, family members, and healthcare professionals can modulate the stress response and enhance the subjective experience of childbirth [32,33,34].

The high intercorrelations reported in previous MSPSS validation studies support the internal consistency of the MSPSS scale, indicating that this instrument validly reflects social support as a relational and multidimensional construct. At the same time, such high intercorrelations suggest that MSPSS primarily captures emotional support, encompassing feelings of being understood, accepted, listened to and surrounded by caring individuals. Although MSPSS does not directly assess instrumental support, this interpretation is based on prior literature [35]. Among the individual sources of support, only partner support showed a significant correlation with positive breastfeeding attitudes, consistent with the broader body of evidence indicating that partner involvement is one of the strongest predictors of breastfeeding initiation, continuation and duration. Partner support may serve as an emotional regulator, reducing stress and enhancing the mother’s sense of security, thereby indirectly fostering positive infant-feeding choices. It may also strengthen parental self-efficacy, shape the perception of childbirth experiences and increase maternal readiness to engage in challenges associated with breastfeeding [25,26,27,28].

In multivariable regression models, perceived social support (MSPSS) did not emerge as a significant independent predictor of breastfeeding attitudes after adjusting for sociodemographic factors. This suggests that its influence may be indirect, operating through other psychological resources—such as childbirth satisfaction, emotional regulation or self-efficacy—rather than exerting a direct effect on feeding attitudes [36]. This pattern was particularly evident in multiparous women, among whom childbirth satisfaction was a stronger predictor than social support. This may indicate that prior childbirth experience and established coping patterns reduce the relative impact of social support on feeding attitudes. Moreover, differences between groups indicate that the influence of social support may be moderated by parity. Among multiparous women, there was a positive correlation between overall perceived support and breastfeeding attitudes, whereas no such association was observed among primiparas. This discrepancy may stem from the fact that first-time mothers frequently experience higher levels of uncertainty, stress and a lack of established cognitive schemas regarding newborn care. In this context, emotional support alone may be insufficient to shape concrete feeding attitudes unless complemented by practical, educational and professional support.

Recent research further highlights that partner support and broader social networks contribute to maternal emotional adjustment and bonding processes after birth, which in turn may shape feeding-related attitudes and behaviors [37,38,39]. Studies show that psychosocial resources—such as perceived support, relationship quality and shared caregiving—are associated with greater maternal well-being and confidence in feeding decisions [40,41,42]. These findings support our interpretation that social support may exert an indirect rather than direct influence on breastfeeding attitudes.

Birth satisfaction was also positively, although modestly, correlated with breastfeeding attitudes and dimensions of social support. This suggests that a positive birth experience, combined with strong relational resources, contributes to a more favorable emotional climate for postpartum adjustment and feeding initiation. These findings align with those noted in studies in which it was shown that women who evaluate their birth experience positively, report higher self-efficacy, more adaptive coping and a lower risk of postpartum depressive symptoms—factors that tend to facilitate breastfeeding engagement and persistence [43,44]. Together, these results highlight that social support—particularly from partners—acts not only as a contextual aid but as a psychological facilitator that shapes attitudes, expectations and early feeding behaviors. According to the Expectancy–Value Model, positive expectations regarding one’s own effectiveness, together with the high value attributed to specific behaviors (e.g., breastfeeding), may contribute to more favorable evaluations of perinatal experiences [45]. Women who enter childbirth with stronger beliefs in their ability to meet maternal challenges may interpret the course of labor more positively, even when faced with difficulties [46].

The regression analyses demonstrated that adding birth satisfaction and perceived social support improved the predictive model only among multiparous women, explaining 19.4% of the variance in breastfeeding attitudes. The effect size (*f*^2^ = 0.143) approached a medium magnitude, suggesting a meaningful but not dominant influence. In contrast, no significant effects were observed in primiparous women, indicating that other psychological or clinical factors not captured in the model may play a more substantial role in shaping their breastfeeding attitudes. This pattern suggests that prior childbirth experience provides an experiential framework that enables mothers to interpret and integrate the birth experience more coherently, translating it into more stable attitudes toward infant feeding. Previous studies have indicated that multiparous women typically exhibit greater emotional regulation, higher maternal self-efficacy, and clearer expectations regarding postpartum demands—all factors that may strengthen the link between birth experiences and subsequent attitudes and behaviors [47,48]. The absence of significant associations among primiparous women may reflect higher levels of uncertainty, fluctuating emotional states, and the complexity of adapting to the maternal role for the first time. Primiparity—especially at advanced maternal age—has been associated with greater anxiety, lower breastfeeding self-efficacy, inconsistent expectations, and increased sensitivity to medical and social influences, which may weaken or obscure the relationship between birth satisfaction and feeding attitudes It is also plausible that additional unmeasured factors (e.g., childbirth fear, medical complications, perceived competence, or informational overload) play a stronger role in shaping attitudes among first-time older mothers. Longitudinal and qualitative studies are needed to investigate these mechanisms more precisely [45].

Our findings align with the Expectancy–Value Model and the Stress and Coping Theory, indicating that cognitive evaluations and stress-regulation processes jointly shape infant-feeding attitudes among women aged ≥35. Consistent with EVM, higher birth satisfaction may strengthen perceived control and reinforce positive appraisals, thereby contributing to more favorable breastfeeding attitudes among multiparous mothers. Concurrently, the Stress and Coping framework clarifies why social support, although not an independent predictor in regression models, may indirectly influence feeding attitudes by mitigating stress and enhancing coping resources. Together, these findings underscore the interplay between evaluative and coping mechanisms in the formation of infant-feeding attitudes in later motherhood.

However, these conclusions must be interpreted with caution. The cross-sectional design precludes causal inference, and the high proportion of highly educated participants may limit generalizability. Furthermore, measurement overlap within the IIFAS-Pol and a reliance on self-reported data introduce additional interpretative constraints. In future research, longitudinal, multicenter and mixed-method designs should be incorporated to more precisely identify causal mechanisms and to explore how women integrate emotional, relational and experiential factors into their feeding decisions across diverse social contexts.

### Strengths and Limitations

This study has several notable strengths. In it, Polish adaptations of validated psychometric instruments were utilized—the Multidimensional Scale of Perceived Social Support (MSPSS), the Iowa Infant Feeding Attitude Scale (IIFAS) and the Revised Birth Satisfaction Scale (BSS-R PL)—which ensured the reliability of the measurements and enabled cross-cultural comparison with other studies. The questionnaire covered a broad range of sociodemographic, obstetric and psychosocial factors, allowing for a multidimensional perspective on late motherhood. The inclusion of both primiparous and multiparous women provided a comparative insight into differences within this population.

However, several limitations should be acknowledged. As a cross-sectional, self-reported survey, causal relationships between birth satisfaction, social support and breastfeeding attitudes cannot be determined via the study. The use of an online questionnaire—while limiting opportunities to clarify ambiguous responses—allowed for efficient recruitment of women from different regions of the country and reduced logistical burden. Previous studies have shown that self-administered online questionnaires can yield data of comparable reliability to those collected in face-to-face settings, particularly when the instruments used are standardized and validated. Another limitation concerns the overrepresentation of women with higher educational attainment. This pattern is common in studies employing open online recruitment, which tend to attract individuals with greater digital literacy, higher health awareness, and more proactive engagement in parenting communities. Women who are digitally active, health-conscious, and involved in online parenting networks were therefore more likely to participate, whereas mothers with limited internet access or lower health literacy may be underrepresented. Although this trend reflects broader demographic shifts observed in Poland [49], particularly among women, it may restrict the generalizability of the findings to populations with lower educational levels and fewer digital resources [50]. Future research should include more socio-demographically diverse groups of women to better reflect the educational and digital diversity of mothers aged ≥35.

The reliance on retrospective self-assessment introduces the risk of recall and social desirability bias. In addition, the absence of detailed clinical or biological data—such as lactation course, obstetric complications or maternal and infant health indicators—prevents examination of physiological factors that may affect breastfeeding outcomes.

Additionally, the study did not include a comparison group of younger mothers (<35 years), which limits the possibility of directly contrasting psychosocial patterns across different maternal age groups.

In interpreting correlations between the key study variables, it is important to consider the possibility of measurement overlapping between certain constructs. The exceptionally strong associations observed between the IIFAS-Pol total score and its breastfeeding and formula-feeding subcomponents (r = 0.917 and r = 0.898, respectively), as well as the positive correlation between attitudes towards breastfeeding and formula feeding, may partially reflect shared content or structural characteristics of the scale rather than exclusively independent psychological domains. Some IIFAS-Pol items—particularly reverse-coded statements—may tap into a general evaluative tendency towards infant feeding rather than capturing distinct attitudinal dimensions. Similarly, modest correlations between social support (MSPSS) and birth satisfaction (BSS-R) may reflect overlapping elements related to perceived emotional support and relational quality. Acknowledging this potential measurement overlap is essential when interpreting the strength and direction of the observed associations, and future studies could benefit from incorporating additional subscales or complementary instruments designed to differentiate nuanced or mixed infant-feeding attitudes. The absence of clinical data (e.g., birth complications, lactation difficulties, neonatal health) may account for the modest strength of the correlations and limits the interpretation of the psychosocial determinants of feeding.

Additionally, several of the statistically significant associations observed in this study were small in size (r ≈ 0.17). While such correlations can reach statistical significance in samples of this size, their practical relevance is limited, and they should be interpreted cautiously. The noted small effect sizes suggest that the examined psychosocial variables explain only a modest proportion of variance in breastfeeding attitudes, indicating that other unmeasured factors—such as personal beliefs, clinical circumstances or cultural influences—may play a more substantial role. Therefore, the current findings should be considered preliminary, and future studies with broader psychosocial and clinical covariates are needed to better understand the determinants of breastfeeding attitudes among women aged ≥35.

Despite these limitations, the study contributes meaningful preliminary evidence on the psychosocial correlates of birth satisfaction and breastfeeding among women of advanced maternal age. The use of standardized tools, adequate sample size and focus on an under-researched population provide a valuable foundation for future longitudinal and mixed-methods research in which psychosocial, medical and biological dimensions of late motherhood could be integrated.

## 5. Conclusions

In this study, it is suggested that among women who become mothers at ≥35 years of age, prior childbirth experience plays an important role in shaping attitudes towards infant feeding. Only in multiparous women did greater birth satisfaction translate into more positive attitudes towards breastfeeding, whereas this pattern was not observed in first-time mothers.

These findings indicate that experiential factors may be particularly relevant for breastfeeding-related attitudes in later motherhood. Women in this age group appeared to approach feeding decisions flexibly, recognizing benefits associated with both breastfeeding and formula feeding. This highlights the importance of individualized, non-prescriptive support that takes personal circumstances and prior birth experiences into account. Interventions aimed at promoting positive birth experiences and strengthening partner involvement may help foster more favorable attitudes towards breastfeeding.

The scope of these conclusions is necessarily limited by the study design and sample characteristics. In future research, longitudinal and mixed-method approaches should be employed, and more diverse populations are needed to clarify causal pathways and better understand how social support, birth experiences and maternal context interact in shaping feeding attitudes at advanced maternal age.

## Figures and Tables

**Table 1:** Study group characteristics.

Category	Variable	Mothers Aged >35 at Birth of Subsequent Child n (%)	Mothers Aged >35 at First Childbirth n (%)
Place of residence	Village Town ≤100,000 inhabitants City >100,000 inhabitants	10 (14.9) 22 (32.8) 35 (52.2)	18 (22.2) 16 (19.8) 46 (58.0)
Education	Primary Secondary Higher	1 (1.5) 17 (25.4) 49 (73.1)	0 (0.0) 14 (17.3) 67 (82.7)
Marital status	Married/in partnership Single Divorced	65 (97.0) 1 (1.5) 1 (1.5)	78 (96.3) 2 (2.5) 1 (1.2)
Economic status	Very good Good Average Poor	17 (25.4) 34 (50.7) 15 (22.4) 1 (1.5)	26 (32.1) 38 (46.9) 17 (21.0) 0 (0.0)
Number of pregnancies	1 2 3 4 or more	2 (3.0) 22 (32.8) 26 (38.8) 17 (25.4)	44 (54.3) 23 (28.4) 8 (9.9) 6 (7.4)
Mother’s age at birth of first child	Under 25 25–34 35–40 41–45 46 or more	21 (31.3) 36 (53.7) 9 (13.4) 1 (1.5) 0 (0.0)	0 (0.0) 0 (0.0) 70 (86.4) 11 (13.6) 0 (0.0)
Mother’s age at birth of last child	35–40 41–45 46 or more	53 (79.1) 14 (20.9) 0 (0.0)	69 (85.2) 11 (13.6) 1 (1.2)
Mode of delivery	Cesarean section Home birth Natural birth Instrumental birth	34 (50.7) 5 (7.5) 28 (41.8) 0 (0.0)	49 (60.5) 1 (1.2) 30 (37.0) 1 (1.2)
Type of feeding	Mixed feeding Formula feeding only Exclusive breastfeeding	17 (25.4) 4 (6.0) 46 (68.7)	22 (27.2) 9 (11.1) 50 (61.7)
Breastfeeding experience	Positive Neutral Negative Did not breastfeed	48 (71.6) 9 (13.4) 9 (13.4) 1 (1.5)	43 (53.1) 14 (17.3) 19 (23.5) 5 (6.2)

**Table 2:** Results of correlation analysis between positive attitudes towards breastfeeding and formula feeding, IIFAS-Pol score, MSPSS and BSS-R PL.

	Mothers Aged >35 Years, Subsequent Child			Mothers Aged >35 Years, Primiparous		
	IIFAS-Pol	BSS-R PL	MSPSS	IIFAS-Pol	BSS-R PL	MSPSS
IIFAS-Pol	-			-		
BSS-R PL	0.396 **	-		0.054	-	
MSPSS	0.249 *	0.249 *	-	−0.021	0.194	-

* *p* < 0.05; ** *p* < 0.01.

**Table 3:** Linear regression model predicting breastfeeding attitudes (IIFAS-Pol) among mothers aged >35 years.

Model	Variables	Mothers Aged >35 Years, Subsequent Child			Mothers Aged >35 Years, Primiparous		
		B	Beta	*p*	B	Beta	*p*
**1**	**Control variables**						
	Education ^a^	1.084	0.044	0.720	6.588	0.231	0.056
	Economic status ^b^	4.367	0.171	0.168	2.151	0.081	0.487
	Delivery mode ^c^	3.619	0.166	0.183	0.020	0.001	0.994
	*R* ^2^	*R*^2^ = 0.069; *R*^2^adj. = 0.025			*R*^2^ = 0.072; *R*^2^adj. = 0.036		
**2**	**Control variables**						
	Education	0.992	0.040	0.731	6.557	0.230	0.061
	Economic status	2.226	0.087	0.467	2.108	0.079	0.504
	Delivery mode	1.277	0.059	0.654	−0.144	−0.007	0.959
	**Independent variables**						
	MSPSS	0.088	0.159	0.199	−0.006	−0.010	0.932
	Birth satisfaction	0.458	0.310	0.025	0.046	0.027	0.827
	*R* ^2^	*R*^2^ = 0.194; *R*^2^adj. = 0.127			*R*^2^ = 0.073; *R*^2^adj. = 0.011		

Note: MSPSS—Multidimensional Scale of Perceived Social Support; IIFAS-Pol—Iowa Infant Feeding Attitude Scale (Polish version); ^a^ 1 = higher education, 0 = secondary/primary education; ^b^ 1 = very good/good, 0 = average/poor; ^c^ 1 = natural birth, 0 = cesarean section; B—unstandardized coefficient; beta—unstandardized coefficient; *p*—*p*-value; *R*^2^—R-squared; *R*^2^adj.—R-squared adjusted.

## Data Availability

The original contributions presented in the study are included in the article; further inquiries can be directed to the corresponding author.

## Abbreviations

The following abbreviations are used in this manuscript:

MSPSS	Multidimensional Scale of Perceived Social Support
IIFAS	Pol Iowa Infant Feeding Attitude Scale Polish adaptation
BSS-R PL	Revised Birth Satisfaction Scale Polish adaptation
WMA	World Medical Association
